# Random duodenal biopsy to exclude coeliac disease as a cause of anaemia is not cost-efective and should be replaced with universally performed pre-endoscopy serology in patients on a suspected cancer pathway

**DOI:** 10.1007/s10151-018-1756-7

**Published:** 2018-02-23

**Authors:** P. J. J. Herrod, J. N. Lund

**Affiliations:** 1 Department of Surgery, Royal Derby Hospital, Derby, DE22 3NE UK; 2 Division of Medical Sciences and Graduate Entry Medicine, Royal Derby Hospital, University of Nottingham, Derby, UK

**Keywords:** Celiac disease, Serologic tests, Endoscopy, Anaemia, Iron deficiency, Biopsy, Colorectal cancer

## Abstract

**Background:**

Random duodenal biopsy to exclude coeliac disease during upper gastrointestinal endoscopy for the investigation of iron deficiency anaemia remains a common procedure, but is expensive and time-consuming. Serological investigation for coeliac disease is also recommended, having excellent accuracy with the added benefit of lower cost. This study sought to examine the utility of duodenal biopsy and coeliac serology in the diagnosis of coeliac disease.

**Methods:**

A prospectively maintained database was interrogated to identify all patients having upper gastrointestinal endoscopy for the investigation of anaemia between January 01, 2016, and December 31, 2016.

**Results:**

Of the 1131 patients having an endoscopy, coeliac serology was measured in only 412 (36%) and was positive in 9 cases (2%), leading to 6 histological diagnoses of coeliac disease and 3 false positives. Two-hundred and seventy-four patients with negative serology had biopsies taken which were all negative. Only 2/451 (0.4%) patients who had biopsies performed in the absence of a serology test were histologically positive for coeliac disease. The cost per diagnosis of a case of coeliac disease in those with either negative or absent coeliac serology was £18,839 (US$25,244, €21,196).

**Conclusions:**

Random duodenal biopsy is not a cost-effective method of diagnosing coeliac disease and should be replaced with pre-endoscopy coeliac serology.

## Introduction

Investigation of anaemia represents a large workload for endoscopy services [1] with anaemia cited as the primary indication in up to 10% of gastroscopies and 6% of colonoscopies [2]. In 2015, 756,600 upper gastrointestinal (GI) endoscopies were carried out in England alone [3]. In the UK, a large burden of this endoscopy workload falls upon colorectal surgeons as the National Institute for Health and Care Excellence (NICE) recommends referral of patients with iron deficiency anaemia (IDA) to a colorectal clinic under the 2-week wait pathway [4].

Luminal investigation remains the gold standard for investigation of possible malignancy with sensitivities and specificities approaching 100% [5–7]. However, upper GI cancer is one of the rarer causes of IDA with as few as 2% of luminal investigations finding malignancy as the cause [8].

Other upper GI causes of anaemia include use of nonsteroidal anti-inflammatory drugs, peptic ulceration and peptic erosions, the majority of which can be detected through visual assessment of the mucosa alone [9]. Coeliac disease is also a common cause of IDA, often diagnosed by demonstration of microscopic features of the disease in duodenal biopsy [10]. Macroscopic features are mostly absent [11, 12], and so biopsy of normal looking mucosa is recommended by the British Society of Gastroenterology and other bodies in order to exclude coeliac disease as a cause of IDA [10, 13, 14].

Highly sensitive and specific immunological assays for the diagnosis of coeliac disease have been developed during the past 2 decades, and the use of the coeliac serology is recommended in the investigation of IDA [15]. The tests are now so reliable that some authors suggest that biopsy is not always necessary for the diagnosis of coeliac disease [16, 17].

Despite these developments, it remains usual for endoscopists to take random duodenal biopsies in the investigation of IDA to exclude coeliac disease as a cause based on traditional practice and content of guidelines [10, 13]. However, duodenal biopsy carries potential morbidity and mortality [18, 19] and increases financial costs due to biopsy equipment, specimen processing and reporting time. This study aimed to evaluate the utility and cost of random duodenal biopsies compared to serology in the diagnosis of coeliac disease in patients being investigated for IDA in one teaching hospital in the UK.

## Materials and methods

This study was carried out as a service evaluation in a large teaching hospital in the UK, which performs over 9000 upper GI endoscopies each year.

All patients having upper GI endoscopy between January 01, 2016, and December 31, 2016, for investigation of anaemia were identified via a prospective electronic database.

Results of haematological, biochemical and immunological investigations performed prior to endoscopy were obtained from the hospital pathology computer database. Similarly, results of duodenal biopsy were retrieved from the pathology records. Internal National Health Service costs for the processing of serology, histology and biopsy consumables were obtained from the hospital finance department.

Data were analysed using Microsoft Excel (version 2016, Microsoft, USA).

## Results

In total, 1150 upper GI endoscopies were carried out for the investigation of anaemia in the 2016 calendar year on 1131 patients. Only 886/1131 patients investigated for anaemia had microcytosis (MCV < 88.0) and only 159 patients had their serum ferritin measured in the investigation of their anaemia. Fifteen patients had an upper GI endoscopy for investigation of anaemia with no evidence of their serum haemoglobin concentration being below 135 g/l at any time.

**Fig. 1:**
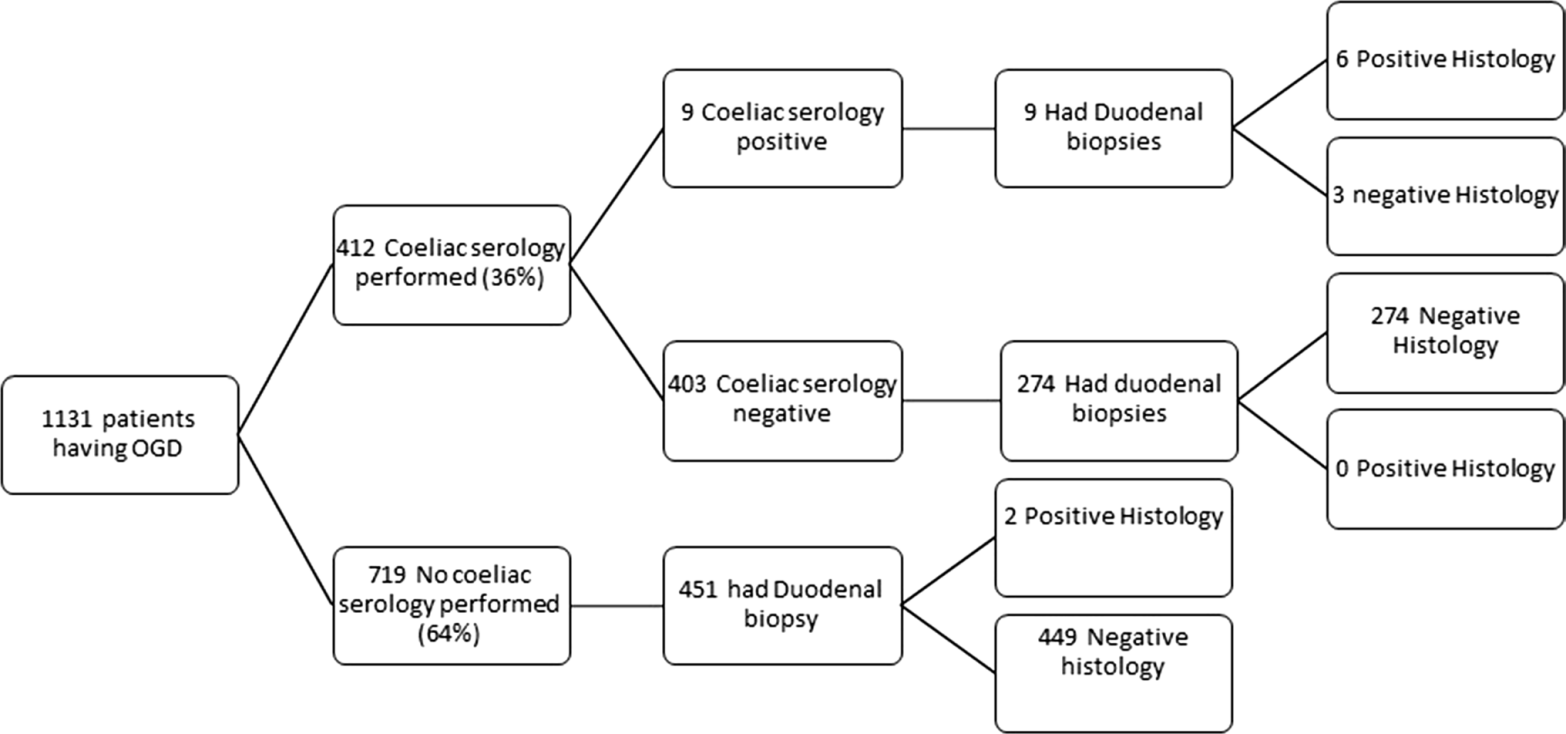
Strengthening the reporting of observational studies in epidemiology flow diagram demonstrating clinical course of patients having an upper gastrointestinal endoscopy

Four hundred and twelve patients (36%) had coeliac serology performed prior to their endoscopy. Seven hundred and thirty-four patients (65%) had duodenal biopsy. A Strengthening the Reporting of Observational Studies in Epidemiology (STROBE) flow diagram demonstrating the clinical pathway followed by all patients having an upper GI endoscopy is displayed in Fig. 1.

Coeliac serology was positive in 9/412 patients (2.2%) in whom it was measured before the procedure. All 9 of these also had duodenal biopsy performed at endoscopy, which was positive for histological markers of coeliac disease in 6 cases and was negative in 3 cases. Of the 403 patients with negative serology, 274 (68%) had duodenal biopsy, none of which demonstrated coeliac disease (249 biopsies reported as normal and 18 as non-specific inflammatory change). Coeliac serology therefore yielded 3 false positive results and no false negatives making the test 100% sensitive and 99% specific.

Of the 719 patients without pre-procedure coeliac serology, 451 had duodenal biopsy (63%), 2 were positive for coeliac disease (0.4%), non-specific inflammatory changes were reported in 37, and the remaining 394 patients had biopsies reported as normal.

The internal National Health Service cost price for the processing of an IgA TTG (tissue trans-glutaminase) titre (the serological test used in our institution) is £6.54 (US$8.73, €7.36). The cost for consumables used in duodenal biopsy is £4.75 for each patient and the cost of processing and reporting each duodenal biopsy £47.22 ($63.06, €53.13).

The total cost of processing the duodenal biopsies in our institution in one calendar year for the investigation of anaemia was £38,146 ($50,943, €42,934). £37,678 was spent processing biopsies from patients who were either TTG negative or had not had their TTG measured, leading to the detection of coeliac disease in 2 patients who had not had TTG performed: a cost of £18,839 ($25,244, €21,196) per case. The cost of measuring TTG in the 719 patients who did not have it performed would have been £4702 ($6278, €5290).

## Discussion

This study suggests that random duodenal biopsy is not cost-effective for the diagnosis of coeliac disease as a cause of IDA and should be abandoned in favour of immunological tests. Whilst serology will not identify all cases of coeliac disease, these tests are 96–98% sensitive and 95–99% specific [20]. In our 1 year of investigation of IDA, 2 patients with coeliac disease who did not have pre-procedure serology were identified by biopsy. The extra cost of identifying each of these cases using a non-selective duodenal biopsy policy was £18,839 per case, far in excess of costs of diagnosing colorectal cancer in practice (between £7586 and £9663 per patient) [21] and is difficult to justify in any financial climate [22, 23]. Indeed, it is almost certain, given the sensitivity of the immunological test, that these cases would have been identified by serology, had it been performed before endoscopy.

With cheap and reliable serological tests, coeliac disease can be excluded without duodenal biopsy [16, 17]. Our experience suggests that performing biopsy on those with negative TTG is unnecessary and more expensive, and although none were seen in our patients, others report a small risk of complications associated with biopsy [18, 19].

The increased costs of biopsy over serology may be underestimated in this study as we have not costed clinical administration time, consumables and postage involved in communicating the result of the biopsy to patient and general practitioner, nor the delay in informing the patient of the outcome of the biopsy. The result of a pre-endoscopy TTG test performed on referral for endoscopy would be ready for patient and endoscopist at the time of the endoscopy and would help to rationalise biopsy practice. Insufficient work-up for IDA remains an issue in this group of patients [24]. Although there were no false negative serology results in our year-long series, continued anaemia after iron therapy or an ongoing suspicion of a potential missed diagnosis of coeliac disease should prompt random duodenal biopsy even if serology is negative, as advised by NICE [13].

## Conclusions

Random duodenal biopsy to exclude coeliac disease as a cause of anaemia is not cost-effective and should be replaced with universally performed pre-endoscopy TTG.
